# The Ethics of the Sewage Question

**Published:** 1886-11-20

**Authors:** 


### The Ethics of the Sewage Question.

General Considerations.

Under every form of civilisation, the most rudimentary or the most advanced, there are certain problems which are always to the front, and always imperiously press for solution. Of these the disposal of sewage is one. The great legislator Moses was a sanitarian, and doubtless his methods were learnt from the practical philosophers of ancient Egypt. The disposal of sewage, meaning thereby all the waste of every kind which is incidental to the life of a city, is one of the crucial tests of a nation's capacity for progression and survival. The crowding of human beings together in large numbers tends to lower their vitality and to diminish their robustness. If no effective measures be taken to check this tendency, its constant operation will inevitably produce the result of diminished capacity for successful struggle, and consequent liability to suppression or extinction at the hands of less cultured but more muscular races. The history of the world is one long illustration of this almost self-evident proposition. The cultured Greeks yielded to the practical Romans; and the Romans themselves, becoming effeminate, were ruthlessly trampled down by the more energetic men of the North. The venerable civilisations of the East, with their polite arts and metaphysical subtleties, have yielded to the sterner physique of the Angles, and the Normans, and the Danes. What is the significance of this to us who live in England, and especially in London? The lesson taught by all these stories and events is this: "Preserve your physical robustness, or you will lose your national existence." No nation can permanently survive by intellect alone. One of the prime factors in the preservation of a people's physical vigour is the wise disposal of their waste products, and the securing of pure air and water in consequence. If sewage be conducted great distances in drains, where it can decompose and escape into the atmosphere as foul gas, or if it be immediately poured into running streams, you either vitiate the air or poison the water drunk by the community. In England both these follies are committed, and with disastrous results. True—by comparison with continental peoples, we are *perfect* sanitarians, but we should rather compare ourselves with the standards of perfection than with the filthy municipalities of continental towns. We are not so bad as we might be, nor are we so good as we ought to be.

The Southampton System.

An interesting event for sewage experts took place recently at Southampton. Mr. Bennett, the able and practical borough engineer, has vigorously endeavoured to secure for the town and seaboard a wholesome atmosphere and pure salt breezes. Yachting men have anything but delightful recollections of the odours of Southampton waters. At low tides the air was poisonous with the foul gases of tons of decomposing sewage which were then thrown into the sea. But the Southampton people have changed all that. Their garbage, dust, and other offal they entirely consume in burning destructors. Their sewage they purify by separating the solid from the liquid constituents, and completely deodorising the latter. The solid portions, after precipitation, are conveyed by pipes to the neighbourhood of the destructor and there mixed with ashes produced by the burning of the garbage. So mixed, the ashes and semi-solid sewage dry rapidly, giving off no evil odour in the process, and form a granular material which can be put into sacks and carted away to the neighbouring farms for use as manure. Up to the present time all the excrementitious matters have been thus mixed with ashes and sold to the farmers at half-a-crown a load. If at any time the demand should fall off, the "sludge," as it is technically called, can be mixed with the garbage and completely burned in the destructors. The material used for the precipitation and separation of the solid from the liquid sewage is "porous carbon," supplied by the London Porous Carbon Company. So far, the series of experiments conducted by Mr. Bennett, ably seconded by the borough analyst, Dr. Arthur Angell, have been signally successful. If similar experiments performed in other towns, as they doubtless will be, should prove equally satisfactory, a step forward will have been taken in practical sanitation, such as has not been witnessed for many generations.

